# Exogenous S1P Exposure Potentiates Ischemic Stroke Damage That Is Reduced Possibly by Inhibiting S1P Receptor Signaling

**DOI:** 10.1155/2015/492659

**Published:** 2015-10-20

**Authors:** Eunjung Moon, Jeong Eun Han, Sejin Jeon, Jong Hoon Ryu, Ji Woong Choi, Jerold Chun

**Affiliations:** ^1^Laboratory of Neuropharmacology, College of Pharmacy and Gachon Institute of Pharmaceutical Science, Gachon University, Yeonsu-gu, Incheon 406-799, Republic of Korea; ^2^Department of Oriental Pharmaceutical Science, College of Pharmacy, Kyung Hee University, Seoul 130-701, Republic of Korea; ^3^Department of Molecular Biology, Dorris Neuroscience Center, The Scripps Research Institute, La Jolla, CA 92037, USA

## Abstract

Initial and recurrent stroke produces central nervous system (CNS) damage, involving neuroinflammation. Receptor-mediated S1P signaling can influence neuroinflammation and has been implicated in cerebral ischemia through effects on the immune system. However, S1P-mediated events also occur within the brain itself where its roles during stroke have been less well studied. Here we investigated the involvement of S1P signaling in initial and recurrent stroke by using a transient middle cerebral artery occlusion/reperfusion (M/R) model combined with analyses of S1P signaling. Gene expression for S1P receptors and involved enzymes was altered during M/R, supporting changes in S1P signaling. Direct S1P microinjection into the normal CNS induced neuroglial activation, implicating S1P-initiated neuroinflammatory responses that resembled CNS changes seen during initial M/R challenge. Moreover, S1P microinjection combined with M/R potentiated brain damage, approximating a model for recurrent stroke dependent on S1P and suggesting that reduction in S1P signaling could ameliorate stroke damage. Delivery of FTY720 that removes S1P signaling with chronic exposure reduced damage in both initial and S1P-potentiated M/R-challenged brain, while reducing stroke markers like TNF-*α*. These results implicate direct S1P CNS signaling in the etiology of initial and recurrent stroke that can be therapeutically accessed by S1P modulators acting within the brain.

## 1. Introduction

Cerebral ischemia produced during stroke is triggered by sudden lack of blood flow and subsequent reperfusion of the ischemic area. Within a few minutes of onset, neurons in the ischemic core are irreversibly injured, which in part determines the fate of brain tissue in the penumbra areas after stroke [1]. Brain damage results from a cascade of cellular and molecular events, including energy failure, excitotoxicity, oxidative stress, and neuroinflammation [2], the latter of which is characterized by CNS infiltration of immune cells and activation of neuroglia such as microglia and astrocytes; neuroinflammation also results in the production of a variety of neurotoxic molecules, including proinflammatory cytokines, all of which produce brain damage [1, 3, 4].

Recurrent stroke, which is a common sequel to an initial stroke, leads to worsened patient outcomes and is thought to be a major cause of morbidity and mortality among initial stroke survivors. Neuroinflammation has been associated with an increased risk of recurrent stroke following transient ischemic attack and may contribute to more severe damage [5–10]. Several proinflammatory factors have been reported to be active in recurrent stroke, including IL-6, TNF-*α*, lipoprotein-associated phospholipase A_2_, C-reactive protein, and fibrinogen [9, 11–14].

Another molecule implicated in neuroinflammation is the lysophospholipid known as sphingosine 1-phosphate (S1P), produced by the phosphorylation of sphingosine by two kinases, sphingosine kinases 1 and 2 (SPHK1 and SPHK2) [15], which acts predominantly as an extracellular signaling molecule through 5, cognate G protein-coupled receptors [16]. This lipid signaling system has been extensively studied in neuroinflammatory processes associated with multiple sclerosis (MS) [17–20] through actions on both immune and CNS cells, where reductions in signaling promote therapeutic efficacy [21–26]. In addition to MS, S1P signaling has also been implicated in other CNS pathologies including Sandhoff disease and demyelination [21, 26–30]. Prior reports implicated S1P signaling in cerebral ischemia where it was presumed to act through effects on immune cells, including elevated S1P levels [31] and that the nonselective S1P receptor modulator, FTY720 (fingolimod), a current therapy for MS [17–20], reduces brain damage in cerebral ischemia [32–36]. Intriguingly, fingolimod improved outcomes in a proof-of-concept clinical trial of 23 patients with intracerebral hemorrhage at both acute (days) and chronic (months) time points [37], consistent with S1P signaling effects in human stroke.

In this study, we have assessed the possibility of direct CNS S1P receptor signaling in M/R models of stroke and focusing on changes occurring within the brain. We report that local increases in S1P within the brain potentiate damage produced by transient focal cerebral ischemia (M/R), which may represent a new model for recurrent stroke, particularly in view of the effects on markers like TNF-*α*. Importantly, we report that S1P receptor modulation using FTY720 can reduce stroke damage in both primary and recurrent stroke models.

## 2. Materials and Methods

### 2.1. Animals

**Figure 1:**
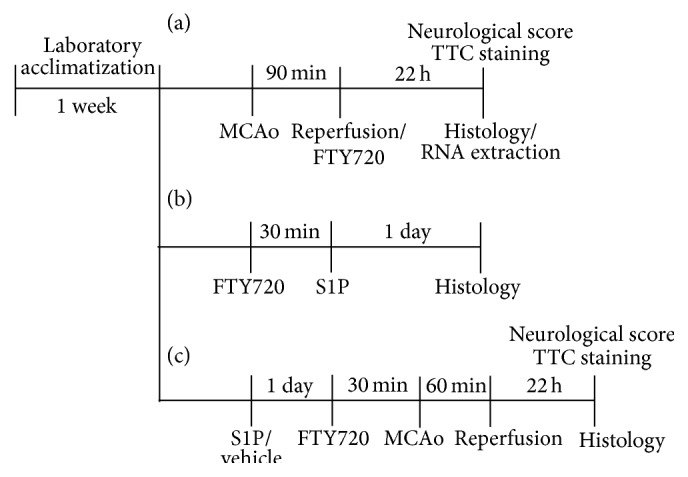
Schematic representation of experimental protocols. (a) Scheme for Figures 2 and 4 (an initial stroke model). Mice were challenged by 90 min occlusion followed by 22 h reperfusion. FTY720 (3 mg/kg, i.p.) was administered to mice immediately after reperfusion. (b) Scheme for Figure 3. S1P was microinjected into the corpus callosum (CC) and brain samples were prepared 1 day after microinjection. FTY720 was administered 30 min prior to S1P microinjection. (c) Scheme for Figures 5 to 7 (a recurrent stroke-mimicking model). S1P was at first microinjected into the CC. One day later, mice were challenged by 60 min occlusion followed by 22 h reperfusion. FTY720 (3 mg/kg, i.p.) was administered to mice 30 min prior to MCAO.

All animal experiments were conducted in accordance with the Center of Animal Care and Use (CACU) guidelines of Lee Gil Ya Cancer and Diabetes Institute (LCDI) at Gachon University (numbers of approved animal protocols: LCDI-2012-0075 and LCDI-2014-0016). Adult male ICR mice (28–32 g, 7 weeks old) were purchased from the Orient Co., Ltd. (Korea), and were housed under controlled temperature (22 ± 2°C), constant humidity, and a 12 h light/dark cycle (light on 07:00–19:00), with food and water made available* ad libitum*. After S1P microinjection or middle cerebral artery occlusion (MCAO) and reperfusion (M/R) surgery (Figure 1, experimental scheme), mice were housed 4 per cage with moist food and soft bedding materials to reduce suffering until they were sacrificed by CO_2_ inhalation or used for sampling.

### 2.2. Materials

S1P [D-erythro-sphingosine-1-phosphate] was purchased from Avanti Polar Lipid (Alabaster, AL). FTY720 [2-amino-2-[2-(octyl-phenyl) ethyl]-1,3-propanediol hydrochloride] was kindly provided by Novartis AG (Basel). 2,3,5-Triphenyltetrazolium (TTC), 3,3′-diaminobenzidine tetrahydrochloride (DAB), fatty-acid-free BSA (FAF-BSA), mouse monoclonal anti-glial fibrillary acidic protein (GFAP) antibody, anti-*β*-actin antibody, cresyl violet acetate, and protease inhibitor cocktail were purchased from Sigma-Aldrich (St. Louis, MO). Silicon (Variotime) and Zoletil 50 were obtained from Heraeus Kulzer GmbH (Germany) and Virbac (Carros, France), respectively. Goat polyclonal anti-Iba1 and rabbit polyclonal anti-TNF-*α* antibodies were purchased from Abcam (Cambridge, UK). Avidin-biotin-peroxidase complex (ABC) kit and Vectashield were purchased from Vector Laboratories, Inc. (Burlingame, CA). Fluoro-Jade B was purchased from Chemicon (Temecula, CA).

### 2.3. Microinjection of S1P at Corpus Callosum (CC)

S1P was dissolved in DMSO with 1 N HCL (95 : 5 v/v, 20 mM) and diluted in 10% FAF-BSA to make a stock (2 mM; 1 nmole/0.5* μ*L). S1P (1 nmol/0.5 *µ*L dissolved in 10% FAF-BSA) was injected at 0.1 *µ*L/min into the right CC of mice anesthetized with the mixture of Zoletil 50 (10 mg/kg, i.m.) and Rompun (3 mg/kg, i.m.). Stereotaxic coordinates were as follows: AP (anteroposterior) = +0.9 mm anterior to bregma, ML (mediolateral) = ±1.0 mm, and DV (dorsoventral) = −2.15 mm. For control mice, 10% FAF-BSA solution containing the same amount of DMSO and HCl was used as vehicle instead of S1P. These mice were used for additional experiments 24 h after microinjection, including M/R challenge (60 min of MCAO) and histological analysis.

### 2.4. Induction of Transient Focal Cerebral Ischemia

M/R-induced focal ischemia was produced by an intraluminal suture method as reported [38, 39]. Briefly, mice were anesthetized with 3% isoflurane in N_2_O and O_2_ (70 : 30) and maintained on 1.5% isoflurane. MCAO was induced by inserting a 9 mm long 5-0 nylon monofilament coated with silicon from the bifurcation to the MCA. In general, blood flow was restored 90 or 60 min after MCAO by carefully withdrawing the monofilament to allow complete reperfusion of the ischemic area under anesthesia. The latter condition (60 min of MCAO) was used to determine damage in recurrent stroke-mimicking situations. Sham-operated animals underwent the same surgical procedure without insertion of nylon monofilament. During the surgery, body temperature was maintained at 37 ± 0.5°C using a heating pad (Biomed S.L., Spain).

### 2.5. FTY720 Administration

FTY720 was dissolved in saline (0.15 M NaCl) and intraperitoneally (i.p.) administered into mice at 3 mg/kg 30 min before S1P microinjection or MCAO surgery to determine its effect on neuroinflammation via S1P exposure or on brain damage by S1P + M/R challenge. Alternatively, FTY720 was administered to mice immediately after reperfusion to determine its therapeutic effect in 90 min M/R-challenged mice. For the control group, an equal volume of saline was administered.

### 2.6. Measurement of Functional Neurological Deficit Score and Infarct Volume

Twenty-two hours after reperfusion, the neurological functions of mice were assessed, including motor function, sensory function, reflex, and balance, using a well-known modified neurological severity score (mNSS), as described previously [39, 40].

Brains obtained 22 h after reperfusion were used to measure infarct volume by staining brain sections (2 mm thickness) with 2% TTC in saline for 30 min. TTC-stained sections were photographed and analyzed using an image J software (National Institute of Mental Health, Bethesda, MD). The infarct volume (%) was calculated for each mouse brain by dividing the lesion volume with the total volume.

### 2.7. Histology

Mice were anesthetized with the mixture of Zoletil 50 (10 mg/kg, i.m.) and Rompun (3 mg/kg, i.m.) and perfused transcardially with ice-cold 50 mM phosphate-buffered saline (PBS, pH 7.4) followed by 4% paraformaldehyde. The brains were removed, postfixed in 4% paraformaldehyde containing 30% sucrose solution (in 50 mM PBS), and frozen with OCT solution. Cryostat sections (20 *µ*m) were used for staining or immunohistochemistry.

For the determination of cell survival or death, cryostat sections were processed for Nissl or Fluoro-Jade B staining as in our previous report [39].

Tissue sections were also used for immunohistochemistry as follows. Tissue sections were treated with 1% hydrogen peroxide in PBS for 15 min, blocked with 5% normal serum containing 0.3% Triton-100, and labeled with primary antibodies, such as goat anti-Iba1 (1 : 500), mouse anti-GFAP (1 : 500), or rabbit anti-TNF-*α* (1 : 100) antibody. The sections were labeled with appropriate biotinylated antibodies (1 : 200) followed by incubation with ABC solution (1 : 100) and then developed with a solution containing 0.02% DAB and 0.01% H_2_O_2_.

Images were taken from each section using a bright-field or fluorescent microscope equipped with a DP72 camera (Olympus Co., Tokyo, Japan). For quantification of immunopositive cells, brain sections of 3~5 mice were analyzed: the number for a mouse brain section was taken after calculating a mean value from 3 images (20x) of each section.

### 2.8. Quantitative Real-Time PCR (qRT-PCR) and Semiquantitative RT-PCR

Total RNA was extracted using RNAiso Plus (Takara) from mouse brain hemisphere subjected to surgical procedure after perfusion with ice-cold PBS and cDNA was synthesized according to the manufacturer's protocols (AffinityScript reverse transcription). qRT-PCR was performed using a Stratagene Mx3005p (Agilent Technologies, Inc., USA) and SYBR Green PCR master mix (Agilent Technologies). Gene expression was quantified using the comparative threshold method and data were calculated as fold changes relative to each gene of sham group after normalization to a reference gene, *β*-actin. Alternatively, 2x master mix (Takara, Japan) was used to conduct semiquantitative RT-PCR. The sequences of all primer sets are listed in Table 1.

### 2.9. Statistical Analysis

All data are presented as mean ± SEM and statistical analysis was carried out using GraphPad Prism software (GraphPad Software Inc., San Diego, CA) as specified. Differences among the groups were analyzed by one-way ANOVA followed by Newman-Keuls test for multiple comparisons. Comparisons between the two groups were performed using paired Student's *t*-test. The statistical significance was set at *P* < 0.05.

## 3. Results

### 3.1. Expression Levels of S1P Signaling-Related Genes Are Altered in M/R-Challenged Mouse Brain

**Figure 2:**
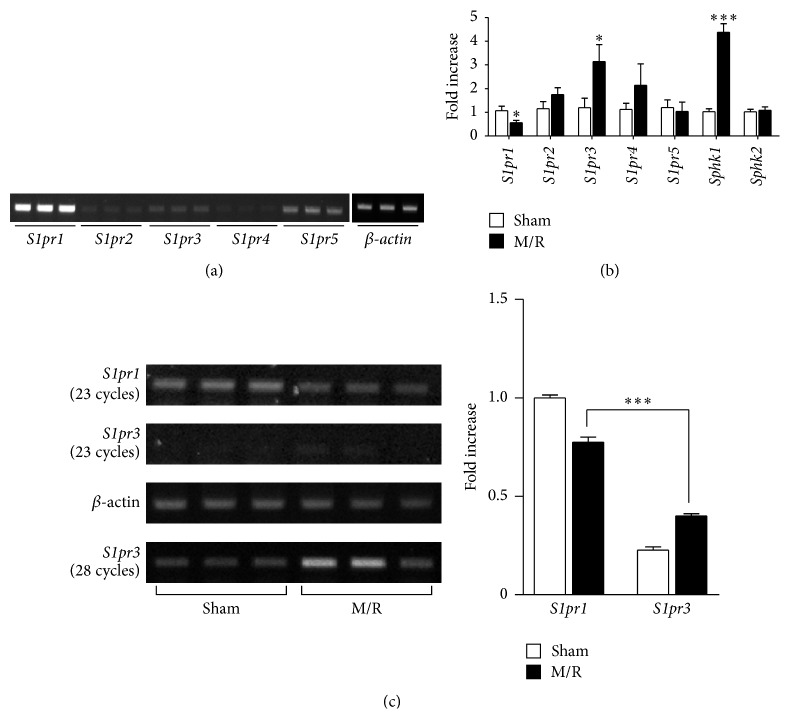
mRNA expression levels of S1P receptors and S1P-producing enzymes are altered in an M/R-challenged brain. (a) Normal brains were used to determine mRNA expression levels of S1P receptors (*S1pr1*,* S1pr2*,* S1pr3*,* S1pr4*, and* S1pr5*) and SPHKs (*Sphk1* and* Sphk2*) based on semiquantitative RT-PCR analysis (28 cycles for all targets). (b, c) Mice were challenged by 90 min occlusion followed by 22 h reperfusion. (b) Brain samples were used to determine changes of S1P receptors and SPHKs based on qRT-PCR analysis. ^*∗*^ *P* < 0.05 and ^*∗∗∗*^ *P* < 0.001, compared with the sham group (*t*-test), *n* = 5 per group. (c) Brains from sham and M/R-challenged mice were used to determine mRNA expression levels of S1P_1_ and S1P_3_ by semiquantitative RT-PCR analysis. Band intensity (bar graph) was calculated as fold increase relative to* S1pr1* level of sham groups after normalization to *β*-actin. ^*∗∗∗*^ *P* < 0.001, compared with* S1pr1* level of M/R group (Newman-Keuls test), *n* = 3 per group.

We examined whether transient cerebral ischemia influences gene expression levels of S1P receptors (S1P_1–5_) and S1P-producing enzymes (sphingosine kinase 1/2, SPHK1/2) within the brain. Temporal changes in S1P receptors and SPHK1/2 gene expression were assessed by qRT-PCR or semiquantitative RT-PCR, 22 h after M/R reperfusion, as compared to *β*-actin controls. In the normal mouse brain, 4 of 5 S1P receptors were expressed, including S1P_1_, S1P_2_, S1P_3_, and S1P_5_, with particularly high expression of S1P_1_ (Figure 2(a)). In M/R-challenged brains, mRNA expression of S1P_3_ and SPHK1 was significantly upregulated compared with sham-operated brains, with differences 3- to 4-fold higher (Figure 2(b)). In contrast, S1P_1_ was downregulated in the M/R group (Figure 2(b)). When semiquantitative RT-PCR analysis was employed, S1P_1_ downregulation was confirmed as observed in data from qRT-PCR analysis. Interestingly, the lowered expression level of S1P_1_ was still higher than the upregulated S1P_3_ in M/R-challenged brains (Figure 2(c)). These results indicate that S1P receptor expression is altered by cerebral ischemia.

### 3.2. Local S1P Microinjection Activates Microglia and Astrocytes

**Figure 3:**
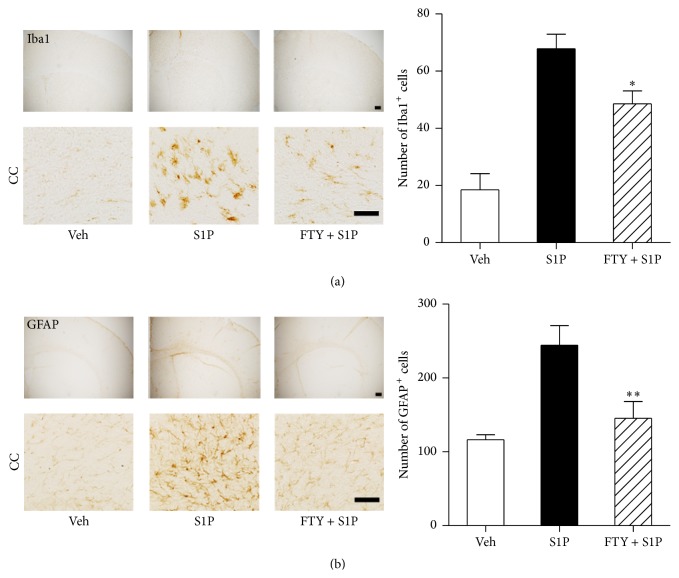
Microglia and astrocytes are activated in the brain following S1P microinjection into the corpus callosum. S1P was microinjected into the corpus callosum (CC), and activation of microglia or astrocytes was assessed 1 day after microinjection. FTY720 (FTY) was administered 30 min prior to S1P microinjection. Representative microphotographs of brain sections immunolabeled against Iba1 (a) or GFAP (b) and their quantitative analysis in groups of vehicle (veh), S1P, and FTY + S1P. ^*∗*^ *P* < 0.05 and ^*∗∗*^ *P* < 0.01, compared with the S1P-injected group (S1P) (Newman-Keuls test). *n* = 5 per group. Scale bar, 200 (upper panel) or 50 *µ*m (lower panel).

Local injection of S1P into the brain induces astrocyte activation [23], which may have relevance to cerebral ischemia in view of changes to S1P signaling molecules. To determine whether direct activation of S1P receptors induces changes in activation of microglia and astrocytes, immunohistochemistry was used to assess the microglia/macrophage-specific marker Iba1 or the astrocyte-specific marker GFAP. S1P microinjection was used to localize S1P at the level of the corpus callosum via defined stereotaxic coordinates (see Section 2) to produce uniform and reproducible exposure. Immunohistochemistry of normal, injected brains revealed increased Iba1-immunopositive cell numbers as compared with vehicle-injected controls (18.50 ± 11.36 to 67.80 ± 11.41: 370%) (Figure 3(a)). In addition, S1P microinjection induced an increase in GFAP-immunopositive cells (116.4 ± 14.91 to 244.4 ± 59.45: 210%) (Figure 3(b)). These neuroinflammatory outcomes were reduced by pretreatment of FTY720 (3 mg/kg, i.p.; Figure 3), a nonselective S1P receptor modulator that acts as a functional antagonist of, at least, S1P_1_ [17, 36]. These results indicate that activation of S1P receptors induces neuroinflammatory changes for neuroglia that can be prevented by pharmacological modulation of S1P receptor activities.

### 3.3. FTY720 Reduces M/R-Induced Brain Infarction and Neuroglial Activation

**Figure 4:**
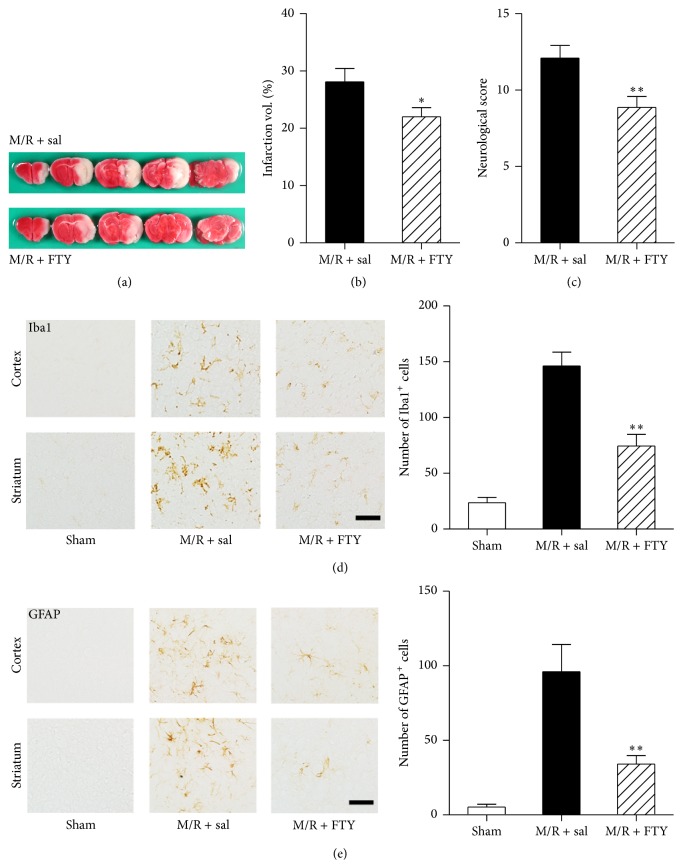
FTY720 reduces brain damage and neuroinflammation in M/R-challenged mice. Mice were challenged by 90 min occlusion and brain infarction or neuroinflammation was assessed 22 h after reperfusion. FTY720 (FTY, 3 mg/kg, i.p.) was administered to mice immediately after reperfusion. (a) Representative TTC-stained brain slices of M/R + saline (sal) and M/R + FTY. Photographs are coronal brain sections stained with TTC showing infarct area (white) and intact area (red). (b) Percentage of infarct volumes calculated from the TTC-stained brain slices. Infarct volume was measured using Image J software, and the percentage of infarction was assessed. (c) Neurological score demonstrating neurological functions. ^*∗*^ *P* < 0.05 (*t*-test), compared with the saline-treated group (M/R + sal) (*t*-test). *n* = 12~15 per group. (d, e) Representative microphotographs of cortex and striatum regions immunolabeled against Iba1 (d) or GFAP (e) and their quantitative analysis in groups of sham, M/R + sal, and M/R + FTY. ^*∗∗*^ *P* < 0.01, compared with the saline-treated group (M/R + sal) of each set (Newman-Keuls test). *n* = 3 per group. Scale bar, 50 *µ*m.

To determine the role of S1P receptor signaling in the pathogenesis of cerebral ischemia, mice were challenged by M/R (90 min of occlusion) and compared to the same challenge except that animals were exposed to FTY720 (3 mg/kg, i.p.) immediately after reperfusion. Brain damage as a percentage of total brain was then assessed by TTC staining of sampled serial sections from the entire brain taken 22 h later (Figures 4(a) and 4(b)). M/R induced brain infarction by 28.08 ± 2.347%, which was reduced by FTY720 administration to 22.00 ± 1.586% (Figure 4(b)). Brain damage was also determined 22 h after reperfusion based on neurological score, showing that FTY720 exposure recovered damaged neurological functions in cerebral ischemia (Figure 4(c)). Neuroglial activation was assessed in M/R groups treated with saline (M/R + sal) or FTY720 (M/R + FTY720) (Figures 4(d) and 4(e)). M/R-challenged mice displayed microglial activation (Iba1-immunopositive cells, Figure 4(d)) and astrogliosis (GFAP-immunopositive cells, Figure 4(e)), which were both markedly decreased in M/R + FTY720 group compared with saline-treated M/R group controls. These data demonstrate that FTY720 significantly decreases brain damage in M/R-challenged mice that is associated with reduced astrocyte and microglial activation, supporting S1P receptor signaling in the brain as a pathological mediator of cerebral ischemia that can be altered to reduce neuroinflammatory changes and brain damage produced by M/R.

### 3.4. Brain Damage Is Augmented by S1P Microinjection

**Figure 5:**
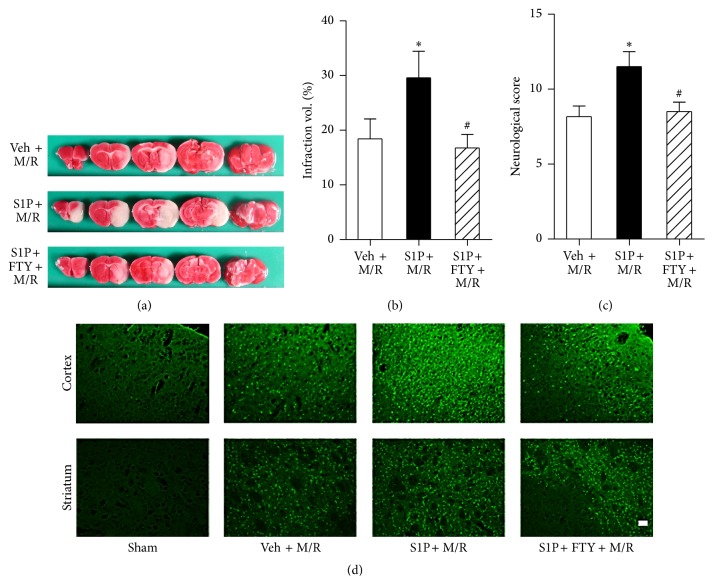
Activation of S1P signaling induces augmented brain damage following M/R injury. S1P or vehicle (veh) was microinjected into the corpus callosum 24 h prior to M/R challenge (60 min occlusion followed by 22 h reperfusion). FTY720 (FTY) was administered into mice 30 min prior to 60 min occlusion. Brain infarction or neuroinflammation was assessed 22 h after reperfusion. (a) Representative TTC-stained brain slices of veh + M/R, S1P + M/R, and S1P + FTY + M/R. Photographs are coronal brain sections stained with TTC showing infarct area (white) and intact area (red). (b) Percentage of infarct volumes calculated from the TTC-stained brain slices. Infarct volume was measured using Image J software and the percentage of infarction was assessed. (c) Neurological score demonstrating neurological functions. ^*∗*^ *P* < 0.05 and ^#^ *P* < 0.05, compared with the M/R group (veh + M/R) and S1P + M/R group, respectively (Newman-Keuls test). *n* = 6~8 per group. (d) Representative microphotographs of cortex and striatum regions stained with Fluoro-Jade B. Scale bar, 50 *µ*m.

Neuroinflammation during initial cerebral ischemia is strongly correlated with recurrent cerebral ischemia, in which more severe brain damage occurs [7–10]. Based on findings that S1P receptors are involved in neuroglial activation and M/R-induced damage, S1P microinjection was used to activate local neuroglia followed by M/R challenge followed by assessments of brain damage. To determine the augmentation clearly, mice were challenged by a shorter M/R (60 min of occlusion and reperfusion) 24 hours after S1P microinjection. S1P microinjection followed by M/R (S1P + M/R) significantly increased damage compared to M/R after vehicle injection (veh + M/R) (Figures 5(a) and 5(b)). Cerebral infarct volume in veh + M/R group was 18.40 ± 3.638% whereas the S1P + M/R group was 29.57 ± 4.867% (Figure 5(b)). This secondary, augmented brain damage produced by initial S1P microinjection was reduced by FTY720 administration prior to M/R challenge (Figures 5(a) and 5(b)). Infarct volume in the S1P + FTY + M/R group was 16.73 ± 2.493 (Figure 5(b)). These results were confirmed by assessments of neurological deficit (Figure 5(c)) and neural cell death using Fluoro-Jade B staining (Figure 5(d)). These data showed that the increased brain damage in S1P + M/R group was blocked by FTY720 administration and indicated that S1P receptor signaling that activates neuroglia—astrocytes and microglia—exacerbates M/R-induced brain damage, possibly representing a model for increased damage observed in recurrent cerebral ischemia.

### 3.5. FTY720 Reduces Neuroglial Activation Occurring in S1P-Primed M/R Challenge

**Figure 6:**
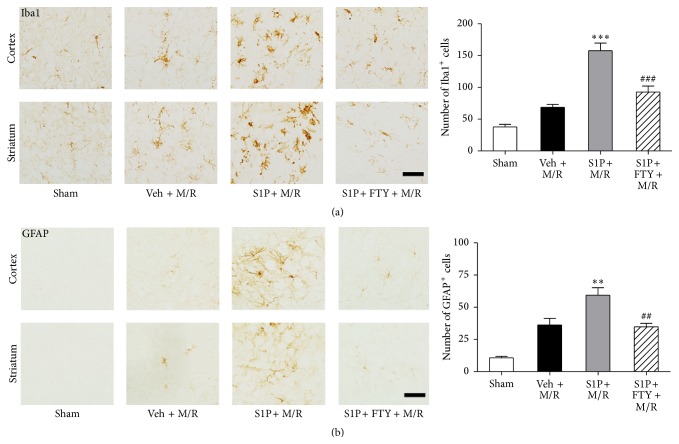
Activation of S1P signaling induces augmented microglial activation and astrogliosis following M/R injury. S1P or vehicle (veh) was injected into the corpus callosum 24 h prior to M/R challenge (60 min occlusion followed by 22 h reperfusion). FTY720 (FTY) was administered into mice 30 min prior to 60 min occlusion. Activation of microglia or astrocytes was assessed 22 h after reperfusion. Representative microphotographs of brain sections immunolabeled against Iba1 (a) or GFAP (b) and their quantitative analysis in groups of sham, veh + M/R, S1P + M/R, and S1P + FTY + M/R. ^*∗∗*^ *P* < 0.01 and ^*∗∗∗*^ *P* < 0.001, compared with M/R group (veh + M/R) (Newman-Keuls test). ^##^ *P* < 0.01 and ^###^ *P* < 0.001, compared with the S1P + M/R group (Newman-Keuls test). *n* = 3 per group. Scale bar, 50 *µ*m.

Local S1P microinjection augmented M/R damage, indicating that CNS S1P receptor signaling potentiates damage produced by ischemic insult. To determine whether local neuroglial activation was also occurring in S1P-primed damage, activated microglia and astrocytes were examined using immunohistochemical markers from animals challenged under various M/R conditions as compared to sham controls. Immunohistochemically observed microglial activation (Iba1-immunopositive cells, Figure 6(a)) and astrogliosis (GFAP-immunopositive cells, Figure 6(b)) were both increased in S1P + M/R group compared to the M/R only group (veh + M/R) and sham controls. The activation was then assessed in animals that had received FTY720 administration prior to M/R challenge: this reduced activation of microglia and astrocytes (S1P + FTY + M/R) (Figure 6).

### 3.6. S1P Microinjection Induces TNF-*α* Expression

**Figure 7:**
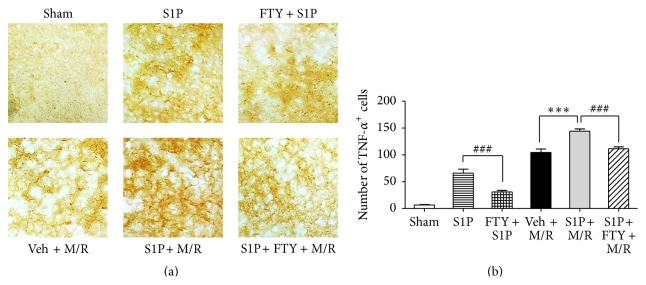
Activation of S1P signaling induces augmented TNF-*α* expression following M/R injury. S1P or vehicle (veh) was injected into the corpus callosum 24 h prior to M/R challenge (60 min occlusion followed by 22 h reperfusion). FTY720 (FTY) was administered into mice 30 min prior to S1P microinjection (FTY + S1P) or 60 min occlusion (S1P + FTY + M/R). Cells expressing TNF-*α* were assessed 1 day or 22 h after S1P microinjection or reperfusion. Representative microphotographs of brain sections immunolabeled against TNF-*α* (a) and their quantitative analysis (b) in groups of sham, S1P, FTY + S1P, veh + M/R, S1P + M/R, and S1P + FTY + M/R. Significance was presented only for the main groups as indicated. ^*∗∗∗*^ *P* < 0.001, compared with M/R group (veh + M/R) (Newman-Keuls test). ^###^ *P* < 0.001, compared with the S1P or S1P + M/R group (Newman-Keuls test). *n* = 3 per group. Scale bar, 50 *µ*m.

The priming of M/R damage by S1P microinjection into the brain was suggestive of changes seen in recurrent stroke, raising the question of whether markers for recurrent stroke might be expressed in the S1P-primed model. Increased TNF-*α* expression is associated with a risk of recurrent stroke [13]. TNF-*α* immunolabeling in the cortex identified significant increases in the number of cells expressing TNF-*α* after S1P microinjection alone (S1P) or M/R alone (veh + M/R) (Figure 7). Notably, the number of TNF-*α*-positive cells was highest in brains challenged by M/R after initial S1P microinjection (S1P + M/R) (Figure 7). All conditions showed a reduction in TNF-*α* expression following FTY720 exposure (FTY + S1P and S1P + FTY + M/R) (Figure 7). These data indicate that S1P receptor-mediated changes can produce pathological changes consistent with recurrent stroke, which can be reduced by modulation of S1P signaling by FTY720 exposure.

## 4. Discussion

The present study has identified activation of S1P receptor signaling within the brain as a factor in transient focal cerebral ischemic (M/R) brain damage, particularly involving activation of astrocytes and microglia. In particular, local brain delivery of S1P—which is independent of M/R, produced astrocyte, and microglial activation—was found to potentiate ischemic brain damage, supporting direct CNS activities of S1P signaling in stroke. FTY720 reduced neuroglial activation and ischemic brain damage and this neuroprotective effect was associated with neuroinflammation [41, 42] wherein neuroglia, such as astrocytes and microglia, are activated by immune cells within the CNS. These data implicate modulation of S1P receptors in forms of stroke, including recurrent stroke, which can be therapeutically accessed by S1P receptor modulation.

Receptor-mediated S1P signaling has previously been suggested to play a role in cerebral ischemia based upon protective effects of the S1P receptor modulator, FTY720. In rodent models of cerebral ischemia, FTY720 reduced ischemic brain damage [32–36], with consistent results observed in a proof-of-concept clinical trial that reported improved neurological endpoints with FTY720 (fingolimod) treatment of brain hemorrhagic stroke patients [37]. The proposed mechanism of FTY720 efficacy in stroke models [36] was similar to that initially proposed for multiple sclerosis (MS) wherein a reduction of pathogenic lymphocytes entering the brain occurs, produced by S1P-dependent alterations of lymphocyte trafficking [18]. This effect is consistent with reported lymphocyte involvement in cerebral ischemia [35].

In addition, evidence for nonimmunological S1P signaling mechanisms occurring within the brain itself has emerged as an explanation for FTY720 efficacy in MS [21, 26–30], which might also be relevant to stroke. Notably, selective removal of the S1P receptor subtype S1P_1_ from astrocytes was found to reduce astrogliosis, disease severity, and FTY720 efficacy in EAE (experimental autoimmune encephalomyelitis), an animal model of MS [26], despite the maintenance of S1P_1_ in the immune system. Reductions in astrogliosis observed here during FTY720 exposure support the operation of a similar protective mechanism involving astrocyte reductions in S1P_1_ signaling. These observations support a direct effect of S1P receptor signaling on the severity of damage produced during stroke, which may involve nonimmunological mechanisms relevant to neuroprotection or repair. Consistent with this possibility, a proof-of-concept clinical trial examining FTY720 effects on hemorrhagic stroke patients reported not only short-term effects that might be immunologically driven, but also longer-term neurological improvement (3 months after event) [37]. Future identification of specific S1P receptor subtypes beyond S1P_1_ and the involved CNS cell types, such as microglia, will assist in elucidating the precise mechanisms of FTY720 efficacy in cerebral ischemia models, which also appears to be relevant to the recurrent stroke model accessed by S1P pretreatment within the brain in view of both damage potentiation by S1P and the increased presence of recurrent stroke markers like TNF-*α*.

Additional support for the importance of S1P signaling mechanisms relevant to stroke may come from studies on S1P itself and its biosynthetic enzymes, SPHK1 and SPHK2. In cerebral ischemia, S1P levels are increased [31] and S1P-producing enzymes may be upregulated in lesion sites [43, 44] even with a contrasting report for the latter [34]. In the normal brain, SPHK2 is more abundantly expressed than SPHK1 [45] and is thought to play a protective role [46]. SPHK2 upregulation in the ischemic or hypoxic brain has been reported [43, 44] but requires further study in view of a contrasting report that SPHK2 was not upregulated [34] and our finding that SPHK1 rather than SPHK2 was upregulated in the ischemic brain. Although this study suggests the importance of SPHK1 in cerebral ischemia based on its mRNA upregulation, a functional role of SPHK1 needs to be clarified. In addition to upregulation of ligand-producing enzyme, our data indicate altered mRNA expression levels of S1P receptors, including S1P_3_ upregulation and S1P_1_ downregulation. It is of note that basal mRNA expression of S1P_1_ is much higher than S1P_3_ both in normal and M/R stroke conditions and that the reduced expression level of S1P_1_ mRNA is still higher than the upregulated level of S1P_3_ in M/R. In fact, there is a report that S1P_1_ is downregulated in the infarcted area 24 h following M/R challenge (2 h MCAo followed by 24 h of reperfusion) in rats possibly through the decreased cerebral blood flow along with ATP depletion in the ipsilateral hemisphere (dead cell regions) [47]. Another study reported the downregulation of S1P_1_ in the kidney 24 h after ischemia/reperfusion [48]. These two independent reports are consistent with our observation that S1P_1_ is downregulated by ischemic challenge. However, the exact mechanism regarding the S1P_1_ downregulation following M/R is still unclear and could be pursued as another study. In addition, it is unclear why FTY720 that is supposed to downregulate S1P_1_ has a protective effect in cerebral ischemia where S1P_1_ is downregulated. Of note, S1P_1_ was also reported to be downregulated at the gene level in rat spinal cords of EAE [49] consistent with overactivation by increased S1P levels, while EAE symptoms were reduced by genetic deletion of S1P_1_ or its functional antagonism by FTY720, consistent with a critical role for the receptor in MS-like disease [26]. These independent results from studies of MS are consistent with our data in M/R stroke models, which also showed astrocyte activation, S1P_1_ downregulation, and the protective effect of FTY720. However, it still remains unclear that S1P receptors with altered expression levels in ischemic brain actually function as pathogenetic factors, which may be tempting to be pursued as a further study.

It is clear that S1P signaling is important for the pathogenesis of cerebral ischemia. This study suggests a possible downregulation of S1P signaling by FTY720, but it is unclear that FTY720 indeed reduces the signaling. It is presumed that the functional antagonism of FTY720 on S1P_1_ may be involved in this neuroprotection. In addition, a recent report demonstrates that FTY720 also causes the downregulation of S1P_3_ [50], as it does for S1P_1_. In this study, S1P_3_ was downregulated at mRNA level in the ischemic brain, so it may be possible that FTY720-mediated functional antagonism on S1P_3_ may contribute to neuroprotection. Therefore, it would be tempting to find pathogenetic role of S1P_1_ or S1P_3_ in cerebral ischemia employing genetic or pharmacological tools to study loss of function in the future.

Neuroinflammation that is featured by the activation of neuroglia, such as microglia or astrocytes, in the brain is an important event contributing to brain damage in both initial and recurrent stroke [1, 3–10]. There are several reports on the anti-inflammatory role of FTY720 in microglial cells [29, 51] through as yet unidentified receptor subtype(s). In activated microglia exposed to lipopolysaccharide, FTY720 reduced activation of inflammation-associated signaling molecules [51] and production of proinflammatory cytokines, such as IL-1*β*, IL-6, and TNF-*α* [29]. In activated astrocytes, FTY720 reduced TNF-*α*-induced ceramide formation [52] even with contrasting results that it did not affect IL-6 production in normal or activated human fetal astrocytes [53]. In this study, FTY720 also reduced neuroglial activation in the ischemic brain. It still remains to elucidate the specific receptor subtypes involved in neuroglial activation. Neuroinflammation is closely linked to blood brain barrier (BBB) disruption and, moreover, S1P signaling has important roles in regulating the BBB, possibly via 2 subtypes of S1P receptors (S1P_1_ and S1P_2_) [54]. It has been reported that S1P signaling modulates BBB integrity, with contrasting roles depending on receptor subtypes: activation of S1P_1_ is linked to enhanced BBB integrity [55–57] while S1P_2_ is linked to increased BBB permeability [58–60]. Moreover, FTY720 was reported to have protective effects on BBB damage through the activation of S1P_1_ [61]. Therefore, the neuroprotective effect of FTY720 observed in this study may be due to its function on the BBB through S1P_1_ because BBB disruption is also pathogenic in the ischemic brain. However, it is of note that S1P-stimulated responses, including S1P microinjection-induced glial cell activation and potentiated brain damage in the ischemic brain, are attenuated by FTY720 exposure. These results suggest that FTY720 might reduce S1P signaling rather than enhance it, but the exact role of S1P receptors, especially S1P_1_, in the ischemic brain still remains unclear.

## 5. Conclusions

Results from the study of both MS and stroke support S1P receptor-mediated signaling as relevant to these diseases through effects not only on neuroinflammation but also through direct CNS effects involving neuroglial activity, with particular relevance to recurrent stroke through the ability of locally microinjected S1P to potentiate M/R stroke damage. Our data are consistent with the primary effects on S1P_1_ and astrocytes; however the role of other S1P receptors and involved cell types in cerebral ischemia awaits future clarification. These data support the actions of S1P receptor modulators for the treatment of stroke, in both the immune system and within the CNS itself, which has therapeutic and mechanistic relevance through targeting these components by brain nonpenetrant versus penetrant compounds.

## Figures and Tables

**Table 1:** Primers used for PCR analysis.

Gene	Direction	Sequence	Gene accession number
*β-actin*	Forward	5′-AGCCTTCCTTCTTGGGTATG-3′	NM_007393
	Reverse	5′-CTTCTGCATCCTGTCAGCAA-3′	

*S1pr1*	Forward	5′-AGGGAACTTTGCGAGTGAG-3′	NM_007901
	Reverse	5′-GTTACAGCAAAGCCAGGTCAG-3′	

*S1pr2*	Forward	5′-ATAGACCGAGCACAGCCAAC-3′	NM_010333
	Reverse	5′-GTGTTCCAGAACCTTCTCAGG-3′	

*S1pr3*	Forward	5′-TTGCAGAACGAGAGCCTATT-3′	NM_010101
	Reverse	5′-TTCCCGGAGAGTGTCATTTC-3′	

*S1pr4*	Forward	5′-ACCTTCAGTCTGCTCTTCACG-3′	NM_010102
	Reverse	5′-AAGAGCACATAGCCCTTGGAG-3′	

*S1pr5*	Forward	5′-AGATTTCCAATAGCCGCTCTC-3′	NM_053190
	Reverse	5′-AGCTTGCCGGTGTAGTTGTAG-3′	

*Sphk1*	Forward	5′-AGTCATGTCCGGTGATGGTC-3′	NM_011451
	Reverse	5′-CCAGTTGGCCTTGGTAGATG-3′	

*Sphk2*	Forward	5′-ATCTCTGAAGCTGGGCTGTC-3′	NM_203280
	Reverse	5′-GAAGAAGCGAGCAGTTGAGC-3′	
